# A cost benefit analysis of varicella vaccination in South Korea

**DOI:** 10.1016/j.jvacx.2024.100521

**Published:** 2024-07-04

**Authors:** Young Hwa Lee, Young June Choe

**Affiliations:** a Allergy Immunology Center, Korea University, Seoul, the Republic of Korea; b Department of Pediatrics, Korea University College of Medicine and Korea University Anam Hospital, Seoul, the Republic of Korea

**Keywords:** Varicella vaccination, Cost-benefit analysis, Disease burden, Korea

## Abstract

**Background:**

The introduction of varicella vaccination has significantly reduced the burden of chickenpox in many countries, but outbreaks still occur in populations with high vaccination coverage. To address this, some countries, including the United States, Germany, and Japan, have adopted a two-dose varicella vaccination recommendation. Economic evaluations are crucial for assessing vaccine recommendations; however, there are limited studies exist in Asian countries. Thus, our study aimed to evaluate the cost-benefit of one-dose and two-dose varicella vaccination programs compared to no vaccination in South Korea, incorporating updated data on disease burden and costs.

**Methods:**

We utilized data from South Korea’s health databases to estimate varicella burden and vaccination records. Decision tree analysis was employed to compare costs and benefits of vaccination strategies over a ten-year period for the 2012 birth cohort. Sensitivity analyses were conducted to assess the impact of various variables.

**Results:**

Both one-dose and two-dose vaccination programs showed cost-benefit compared to no vaccination, with substantial societal cost savings. The one-dose program yielded a benefit-cost ratio (BCR) of1.43, while the two-dose program had a direct BCR of1.28. Sensitivity analyses confirmed the robustness of these findings.

**Conclusion:**

Our study demonstrates the economic benefits of varicella vaccination in South Korea, aligning with findings from other countries. While the second dose did not show additional cost savings compared to the one-dose program, other factors like disease severity and transmission dynamics should be considered. Implementing either a one-dose or two-dose varicella vaccination regimen in South Korea could lead to cost reductions and improved cost-effectiveness compared to no vaccination, emphasizing the importance of vaccination programs in reducing disease burden and enhancing public health outcomes.

## 1 Introduction

Since the introduction of varicella vaccine, the one-dose vaccination program has decreased the burden of chickenpox and its associated costs in many countries [1]. This program achieved a reduction of approximately 83 % in varicella cases compared to the pre-vaccination era, and a 66 % decrease in varicella-related deaths in the United States [2], [3]. Despite these achievements, varicella outbreaks persistently emerge in populations with high vaccination coverage, imposing significant health and economic burdens on public health [4]. To further mitigate the varicella disease burden, countries like U.S. [5], Germany [6], and Japan [7] have endorsed a routine two-dose varicella vaccination recommendation.

Economic evaluations play a pivotal role in assessing vaccine recommendations. Previous cost-effectiveness studies on one-dose varicella vaccination were based on pre-vaccination varicella health burdens, costs, vaccine efficacy, and vaccination coverage [8], [9]. They projected that a fully implemented varicella vaccination program would prevent 93 % of all cases, resulting in net costs to the healthcare system but overall savings for society [8]. Similar results were observed in other US-based studies [9], [10]. A recent study from Mexico concluded that universal varicella vaccination is cost-effective, with strategies involving one or two doses resulting in significant annual savings and reductions in disease burden over a 20-year period [11]. However, these assessments, conducted over different geographic regions, offer limited guidance for policy changes in Asian countries. They assumed higher reductions in disease incidence than observed, lacked updated data on varicella disease burden, vaccine effectiveness, and adverse events post-program implementation.

In South Korea, varicella vaccination has been recommended for children in high-risk groups since 1988 [12]. Subsequently, with the implementation of universal varicella vaccination through the National Immunization Program (NIP) in 2005, a single dose of varicella vaccine has been advised for all children aged 12–15 months. Among the available options, four live attenuated varicella vaccines exist, three of which are based on the Oka strain, while one is based on the MAV strain [1]. Since varicella became a notifiable infectious disease in July 2005, the Republic of Korea transitioned from using the pediatric communicable diseases sentinel surveillance system to a mandatory surveillance system. According to the Infectious Disease Control and Prevention Act, physicians treating varicella patients must report the cases to local public health centers within 24 h [13]. Additionally, public health centers are required to report these cases to the KDCA through municipal or provincial internet-based surveillance systems.

To inform policy decisions, we aimed to evaluate the cost-benefit of both one-dose and two-dose varicella vaccination programs in comparison to the absence of vaccination, incorporating updated disease burden estimates and costs for South Korea. We also analyzed the incremental benefit-cost and cost-effectiveness ratios of the second dose. Ultimately, the objective was to provide decision-makers with evidence-based recommendations to improve varicella control and prevent outbreaks in South Korea.

## 2 Methods

We utilized data from the Health Insurance Review and Assessment Service (HIRA) linked with immunization registry in South Korea. Covering over 98 % of the Korean population, national health insurance operates as a single-payer system, providing comprehensive medical information, including sociodemographic characteristics, from both inpatient and outpatient records nationwide. Claim data diagnosed as varicella (ICD-10, B01) from the Health Insurance Review and Assessment Service (HIRA) database between 2012 and 2022 for the cohort born in 2012 were used to estimate the burden of varicella disease, along with the vaccination records collected from the National Infectious Diseases Surveillance database run by the Korea Disease Control and Prevention Agency (KDCA). The KDCA contributed immunization registry data, encompassing vaccination details, diagnosis dates, series, and vaccine types.

Annual incidence rates and hospitalization rates of the one-dose and two-dose vaccinated and medical treatment costs were calculated from this data, however, the incidence rates of the unvaccinated were obtained from the US study conducted by Zhou et al.(2008) [10], because, after the introduction of the national universal one-dose varicella vaccination program in 2005, there were only a small number of unvaccinated children in South Korea, 12,139 (2.8 %) of the total 484,047, being likely to have unusual medical characteristics or already infected prior to vaccination.

**Fig. 1:**
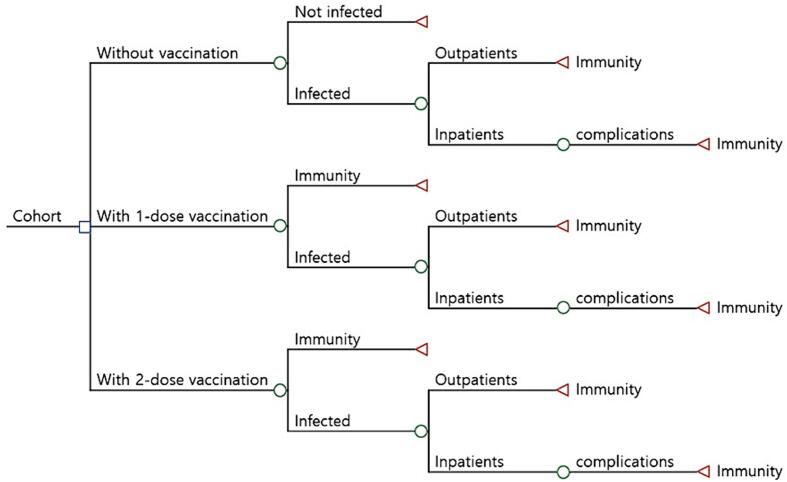
Simplified decision tree.

To compare the costs and benefits between 2022 and 2032 of the three varicella vaccination strategies for the 2022 birth cohort of 277,401 children – no vaccination, one-dose vaccination (at 12–15 months old) and two-dose vaccination (at 4–6 years old), a decision tree analysis was employed (Fig. 1). In the decision tree, a child could be vaccinated one or two times, or not vaccinated. After vaccination, he or she could get infected with varicella according to the effectiveness of vaccine or not. The annual incidence rates by ages assumed in this study were shown in Table 1. If infected, he or she could have moderate symptoms and be treated as an outpatient or could have severe symptoms with complications such as pneumonia, encephalitis, meningitis and keratitis and be hospitalized as an inpatient. The hospitalization rates assumed in this study were shown in Table 2. Direct costs were calculated as costs for services and materials and indirect costs were estimated by using the capital approach – time and wage, along with transportation fares. Outpatient, inpatient, prescription costs were included in medical treatment costs, and these were assumed to be the same in regardless of the vaccination status. Vaccination costs were calculated by using the proportion of administration between public and private sector from our data, due to the difference of procurement costs. These estimated costs were shown in Table 3. Benefits were calculated as the saving costs of reduced varicella cases. All values were evaluated as discounted present value of 2022. The values of time taken for vaccination, inpatient, outpatient and travel, and transport fares were obtained from Korea Health Panel (2008) [14]; wages and economic activity participation rate were from The Economically Active Population Survey (the Ministry of Employment and Labor, 2022) [15]; price indices for health and transport were from The Consumer Price Survey (Statistics Korea, 2022) [16].

**Table 1:** Varicella annual incidence rates with and without vaccination (2012 birth cohort).

Age (year)	Unvaccinated†	1-dose Vaccinated	2-dose Vaccinated
0	55.6	−	−
1	98.4	5.2	3.5
2	98.4	17.3	10.4
3	98.4	19.0	10.0
4	98.4	25.8	10.2
5	81.2	35.7	10.1
6	81.2	37.3	9.3
7	81.2	28.5	8.3
8	81.2	7.3	3.2
9	81.2	3.9	2.3
10	19.1	3.8	1.7
Total	874.3	183.8	69.0

*Data are varicella cases per 1,000 population.

† Data from JID 2008:197 (Suppl 2), Zhou et al.

**Table 2:** Probabilities of hospitalizations rates.

	Unvaccinated	1-dose Vaccinated	2-dose Vaccinated
Outpatients	94.3	98.3	99.1
In patients	5.7	1.7	0.9
Total	100.0	100.0	100.0

**Table 3:** Direct costs and indirect costs for varicella-related events (per case).

		Direct cost	Indirect cost	Total Cost (direct + indirect)
Inpatients				
	Medical treatment cost	832.3	500.3	
Outpatients				
	Medical treatment cost	35.9	43.0	
Vaccine cost a^,^^b^^,^^c^		24.6	15.4	40.0
Public		11.5	12.0	23.5
Private		27.0	16.0	43.0

a Proportion of vaccination facility: public (public health center) 15.1 %, private (hospital, clinic) 84.9 %.

b Exchange rate, 1,250 Korean won for one US dollar.

c Direct vaccination costs were collected from 「Infectious Disease Control And Prevention Act」, Korea Disease Control and Prevention Agency, 2022.8.5. Prive cost consists of public cost and actual medical expense.

Sensitivity analyses were performed to assess the effect on the benefit-cost ratios (BCRs) by varying variables such as vaccine coverage rate, vaccine effectiveness, hospitalization rate, price of vaccine, and discount rate. The ranges of each variable were assumed to reflect the real world in South Korea.

## 3 Results

Estimated costs and benefits for the 2022 birth cohort of 277,401 children over 10 years were summarized in Table 4. If no vaccination program is implemented, there will be 220,222 varicella cases and $15.7 million of social costs while only 46,322 cases and $2.0 million for one-dose vaccination program and 17,359 cases and $0.6 million for two-dose will occur. With one-dose vaccination program amounts for $9.6 million, 173,900 cases would be decreased and $13.7 of the social costs would be saved, leading the total BCR to1.43. With two-dose vaccination program amounts for $17.9 million, 202,863 cases would be prevented and $15.0 million would be saved, leading the total BCR to0.84. The one-dose vaccination program resulted in effective compared to no vaccination program and the two-dose vaccination program was also effective when the direct BCR 1.28 was considered. Two-dose vaccination program showed no additional effectiveness when compared to one-dose vaccination program. If the second dose of vaccine administered, 28,963 cases (62.5 %) would be prevented. Nevertheless, it needs additional vaccination costs of $8.3 million and only would only save $1.3 million of the total costs, bringing the total BCR to 0.16.

**Table 4:** Summary of the costs, benefit-cost ratios (BCRs), and cost-effectiveness ratios.

Variable		Unvaccinated	1-dose Vaccinated	2-dose Vaccinated
Varicella cases, no.		220,222	46,322	17,359
Total costs of varicella, $		15,712,968.9	1,957,278.5	630,242.1
	Direct costs of varicella	14,722,794.1	1,854,636.0	598,534.2
	Indirect costs of varicella	990,174,8	102,642.4	31,707.8

1- or 2-dose vs. unvaccinated				
	Cases prevented, no.		173,900	202,863
	Cases prevented, %		79.0 %	92.1 %
	Cost of vaccination, $		9,603,122.5	17,898,660.8
	Savings in direct costs, $		12,868,158.1	14,124,259.3
	Savings in indirect costs, $		887,532.3	958,467.0
	Savings in total costs, $		13,755,690.4	15,082,726.8
	Direct BCR		2.18	1.28
	Total BCR		1.43	0.84
	Total net present value (net saving), $		4,152,567.9	2,815,934.0
	Cost per case prevented, $		23.9	13.9

2-dose vs. 1-dose				
	Cases prevented, no.			28,963
	Cases prevented, %			62.5 %
	Cost of vaccination, $			8,295,538.3
	Savings in direct costs, $			1,256,101.8
	Savings in indirect costs, $			70,934.6
	Savings in total costs, $			1,327,036.4
	Direct BCR			0.25
	Total BCR			0.16
	Total net present value (net saving), $			−6,968,501.9
	Cost per case prevented, $			−240.6

*Exchange rate, 1,250 Korean won for one US dollar.

Sensitivity analyses for the total BCR for the vaccination strategies showed that almost similar result as the above base case. Being compared with no vaccination program, one-dose vaccination program was cost-benefit, except for cases where vaccine effectiveness drops to 13.0 %, the effectiveness of one-dose varicella vaccine in South Korea [17], or proportion of inpatients drops to 1.7 %. Two-dose vaccination is not cost-benefit than one-dose vaccination in any given conditions. The total BCR of the two-dose vaccination versus no vaccination maintains the range between 0.50 and 1.41; The total BCR could be larger than 1.00 if proportion of inpatients increases or vaccine cost drops, significantly. Vaccine coverage rate and discount rate had little impact on the total BCR (Table 5).

**Table 5:** Sensitivity analysis for relevant parameters.

Variable		Base-case	Range	Total BCR		
				1 vs 0	2 vs 0	2 vs 1
Vaccine coverage rate		100 %	95–100%	1.43–1.51	0.84–0.89	0.16–0.17

Vaccine effectiveness						
	1-dose vaccination	79.0 %	13.0–92.1 %	0.79–1.56	(0.84)	0.01–0.90
	2-dose vaccination	92.1 %	79.0–99.0 %	(1.43)	0.78–0.87	0.03–0.23

Proportion of inpatients						
	Unvaccinated	5.7 %	1.7–10.0 %	0.79–2.12	0.50–1.21	(0.16)
	1-dose vaccination	1.7 %	0.9–3.0 %	1.39–1.46	(0.84)	0.13–0.21
	2-dose vaccination	0.9 %	0.5–1.7 %	(1.43)	0.84	0.15–0.17
Vaccine cost, $		41.5	24.0–56.0	1.02–2.39	0.60–1.41	0.11–0.27
Discount rate		5 %	3–7 %	1.35–1.52	0.82–0.87	0.16

## 4 Discussion

This study highlights the substantial societal cost savings and high benefit-cost ratios associated with both one-dose and two-dose varicella vaccination programs compared to no vaccination program in South Korea. The one-dose program yields approximately $13.7 million in societal cost savings, with a benefit-cost ratio of about $1.43 saved for every dollar spent. Similarly, the two-dose program results in approximately $15.0 million in societal cost savings, with a direct benefit-cost ratio of $1.28 saved for every dollar spent. These findings demonstrate the favorable benefit-cost ratio of varicella vaccination programs, achieving significant public health benefits by reducing morbidity associated with varicella, while also generating substantial cost savings. Our research aligns with economic analyses conducted in the U.S., indicating that compared to no vaccination, both the one-dose program (with a societal BCR of 4.37) and the two-dose program (BCR of 2.73) are estimated to be cost-saving from a societal perspective; however, when compared to the one-dose program, the incremental addition of a second dose does not yield cost savings (societal incremental BCR of 0.56) [10]. The findings may vary depending on the country's epidemiological context, healthcare expenses, consideration of herpes zoster impact, and the type of vaccine formulation (e.g., standalone varicella vaccine versus combination with measles-mumps-rubella vaccine). Research conducted in the UK suggests that introducing a 2-dose universal varicella vaccination is a cost-effective option compared to no vaccination, despite differences in healthcare costs compared to the US, and the use of the MMR-V combination vaccine [18]. Although South Korea has low medical care costs [19], and is densely populated [20], and despite the absence of MMR-V formulations, our results should be interpreted within the context of the country when compared to others. Nevertheless, the findings indicate a net economic benefit of the varicella program.

While the economic analysis indicates that the second-dose varicella vaccination was not cost-saving compared to the one-dose program, the decision to recommend a routine two-dose vaccination program in the U.S. considered various factors beyond cost-effectiveness [10], [21]. These included ongoing disease burden, transmission dynamics, the potential for severe disease and transmission to high-risk individuals, as well as the demonstrated efficacy of the second dose in enhancing immunity and disease protection [22], [23], [24]. Such factors should be also considered in the Korean context. A national cohort study conducted in Korea evaluated the efficacy of one-dose and two-dose varicella vaccination against laboratory-confirmed varicella [25]. The study revealed a vaccine effectiveness (VE) of 16.8 % for one-dose vaccination, while two-doses showed a VE of 98.6 %. In another study, within the 2011 birth cohort comprising 421,070 newborns, one-dose vaccination demonstrated a VE of 86.1 % in the first year and 49.9 % over a 6-year follow-up, indicating a 7.2 % annual decline [26]. A dynamic transmission model, calibrated using age-specific varicella incidence data and vaccine coverage in Korea, revealed moderate effectiveness of the single-dose vaccine [27]. In the scope of vaccinology, where primary failure played a crucial role in breakthrough infections among children who received only one dose, it is essential to discuss the potential benefits of implementing a two-dose vaccination strategy for public health purposes. A 2012–2013 cross-sectional seroepidemiological study in Korea found that while overall varicella seroprevalence was high, it was significantly lower in adolescents aged 10–19 compared to older age groups, highlighting the need for strategies to enhance varicella immunity in teenagers. [28].

This study had several limitations. Due to the lack of varicella incidence data before the introduction of the national vaccination program, such as the incidence rates of the unvaccinated, that this study may not fully reflect the exact epidemiologic conditions of the unvaccinated in Korea. Furthermore, the change in the varicella vaccine distributed in Korea was not considered. The vaccine used mainly in the 2012 birth cohort used in this study is not currently distributed. As a result, there may be changes in incidence and hospitalization rates due to the use of a different vaccine from the past. Moreover, it is important to acknowledge that our analysis does not account for potential vaccine adverse reactions, which may impact the overall cost-effectiveness assessment. In addition, we acknowledge the potential for underreporting in our study, which may affect the accuracy of our estimates and should be considered when interpreting the results. A study found that the recent increase in reported varicella cases is influenced by improved reporting after varicella was included in the NIP system [29]. Despite these limitations, our study provides valuable insights into the costs and benefits of such a strategy. While our model may serve as a template for other countries considering varicella vaccination programs, it's crucial to adapt the analysis to each country's unique healthcare delivery setting and epidemiological context. Our sensitivity analysis underscores the importance of discount rates and vaccine prices in determining the cost-benefit of the second dose. Overall, the findings underscore the importance of vaccination in reducing the burden of varicella disease and improving public health outcomes.

In conclusion, our findings suggest that implementing either a one-dose or two-dose varicella vaccination – when considering direct BCR – regimen could result in cost reductions and improved cost-benefit compared to no vaccination. This indicates that allocating resources to varicella vaccination programs could result in considerable advantages, both in terms of economic savings and public health outcomes.

## CRediT authorship contribution statement

**Young Hwa Lee:** Writing – review & editing, Writing – original draft, Resources, Project administration, Methodology, Investigation, Funding acquisition, Formal analysis, Data curation, Conceptualization. **Young June Choe:** Writing – review & editing, Writing – original draft, Visualization, Validation, Supervision, Conceptualization.

## Declaration of competing interest

The authors declare the following financial interests/personal relationships which may be considered as potential competing interests: Young June Choe reports financial support was provided by SK Bioscience Co Ltd. If there are other authors, they declare that they have no known competing financial interests or personal relationships that could have appeared to influence the work reported in this paper.

## Data Availability

Data will be made available on request.
